# Erratum: Balconi, M.; et al. Evidences from Rewarding System, FRN and P300 Effect in Internet-Addiction in Young People SHORT TITLE: Rewarding System and EEG in Internet-Addiction *Brain Sciences* 2017, *7*, 81

**DOI:** 10.3390/brainsci7090116

**Published:** 2017-09-11

**Authors:** Michela Balconi, Irene Venturella, Roberta Finocchiaro

**Affiliations:** 1 Research Unit in Affective and Social Neuroscience, Department of Psychology, Catholic University of the Sacred Heart, 20123 Milan, Italy; irene.venturella@unicatt.it (I.V.); roberta.finocchiaro@unicatt.it (R.F.); 2 Department of Psychology, Catholic University of the Sacred Heart, Milan Largo Gemelli, 1, 20123 Milan, Italy

We would like to submit the following erratum to our recently published paper [1] due to the error in the title. We request to change the title to “Evidences from Rewarding System, FRN and P300 Effect in Internet-Addiction in Young People”, and delete “SHORT TITLE: Rewarding System and EEG in Internet-Addiction”.

We apologize for any inconvenience caused to our readers.

## References

[1] Balconi M., Venturella I., Finocchiaro R. (2017). Evidences from Rewarding System, FRN and P300 Effect in Internet-Addiction in Young People SHORT TITLE: Rewarding System and EEG in Internet-Addiction. Brain Sci..

